# miR-513b-5p inhibits the proliferation and promotes apoptosis of retinoblastoma cells by targeting TRIB1

**DOI:** 10.1515/med-2021-0343

**Published:** 2021-09-09

**Authors:** Li-Juan Zhang, Fang Wang, Pei-Yan Qi, Wei-Yan Zhou, Bing Wang

**Affiliations:** Department of Ophthalmology, Shandong Provincial Hospital Affiliated to Shandong First Medical University, Jinan 250021, Shandong, China; Guangzhou International Travel Health Care Center, Guangzhou 510000, Guangdong, China

**Keywords:** miR-513b-5p, TRIB1, Weri-RB1 cells, proliferation, apoptosis, retinoblastoma

## Abstract

MicroRNAs are involved in the pathogenesis of various human malignant tumors. This study aims to explore the role of miR-513b-5p in the malignant proliferation of retinoblastoma (RB) cells and its potential molecular mechanisms. The function-gain and function-loss experiments were performed in Weri-RB1 cells using miR-513b-5 mimics and inhibitors. miR-513b-5p mimics inhibited the proliferation and clone formation and promoted apoptosis of Weri-RB1 cells. In contrast, the miR-513b-5p inhibitor promoted the proliferation and clone formation of Weri-RB1 cells and inhibited cell apoptosis. miR-513b-5p can directly bind to the 3′UTR region of TRIB1 mRNA, and inhibit its protein expression. Overexpression of TRIB1 promoted the proliferation and cloning of Weri-RB1 cells but inhibited their apoptosis. The knockdown of TRIB1 inhibited the proliferation and clone formation of Weri-RB1 cells and promoted cell apoptosis. In addition, miR-513b-5p mimics neutralized the effects of TRIB1 overexpression on the proliferation and apoptosis of Weri-RB1 cells. Finally, miR-513b-5p can inhibit the phosphorylation level of AKT, mTOR, and p70, while TRIB1 played the opposite role. miR-513b-5p inhibits the malignant proliferation of Weri-RB1 cells by repressing the expression of TRIB1. miR-513b-5p and TRIB1 may be the biomarkers and/or key targets for clinical diagnosis and treatment of RB.

## 1 Introduction

Retinoblastoma (RB) is the most common intraocular malignant tumor in infants, which seriously endangers their visual function and life. It accounts for 2.5–4% of malignant tumors in children, with a prevalence of 1/20,000 to 1/15,000 [1,2]. In recent years, with the continuous improvement of the diagnosis and treatment methods, the prognosis and survival rate of RB patients have been improved. However, the pathogenic factors and molecular mechanisms of the disease have not been fully studied. Therefore, further research and exploration of genes and action mechanism that related to the occurrence and development of RB are of great significance [3].

MicroRNAs (miRNAs) are a group of endogenous non-coding RNA molecules with a length of 20–22 nucleotides [4]. They can directly interact with the 3′-non-translation regions (3′UTR) of the target mRNA, leading to mRNA degradation or translation inhibition, thereby negatively regulating the expression of the target genes [5]. miRNAs have an important relationship with the occurrence and development of cancer [6]. Many different miRNA abnormalities have been identified in RB. Their abnormal expression plays an important role in various processes of cancer, including cell proliferation, metastasis, apoptosis, invasion, epithelial–mesenchymal transformation, and angiogenesis [7].

Previous studies have shown that miR-513b-5p can play a unique role in different types of solid tumors. For example, the expression of miR-513b-5p is significantly downregulated in non-small cell lung cancer (NSCLC) [8,9] and gastric cancer [10]. Its exogenous overexpression can significantly inhibit the proliferation, invasion, migration, and promote apoptosis of NSCLC [8,9] and gastric cancer cells [10]. However, some studies have also reported that miR-513b-5p plays an oncogene in cervical cancer [11] and ovarian cancer [12]. In addition, miR-513b-5p has also been identified to play an important role in testicular development and male sexual maturation [13,14]. However, the role of mir-513b-5p in RB generation has not been reported.

The aim of this study was to investigate the effect of miR-513b-5p overexpression and downexpression on the proliferation and apoptosis of RB cells and its possible mechanism. The results may provide some new insights into the pathogenesis of RB and help to explore new treatments.

## 2 Materials and methods

### 2.1 Cell culture

The retinoblastoma cell line Weri-RB-1 and 293 T cell were obtained from the American Type Culture Collection (ATCC), and cultured in the Dulbecco’s modified Eagle’s medium (DMEM; Gibco; Thermo Fisher Scientific, Inc.) with 10% FBS, 100 U/mL penicillin, and 0.1 mg/mL streptomycin at 37°C with the atmosphere of 5% CO_2_.

### 2.2 Cell transfection

miR-513b-5p mimics, miR-513b-5p inhibitor, siRNA specifically targeting TRIB1 (TRIB1-KD), TRIB1 overexpression plasmid (TRIB1-OE), and their respective negative control (NC) were obtained from GenePharma Co. Ltd. (Shanghai, China). Transfection was performed according to the instructions of the Lipofectamine2000 transfection kit (Invitrogen; Thermo Fisher Scientific, Inc.).

### 2.3 Cell Counting Kit‑8 (CCK-8) assay

The transfected cells were seeded into a 96-well plate at a concentration of 1 × 10³ cells per well. After culturing for 24, 48, and 72 h, the old medium in each well was removed, and fresh DMEM containing 10% CCK8 reagent (Beyotime Institute of Biotechnology) was added and incubated at 37°C for 2 h. Subsequently, the absorbance value (OD) of each well was detected with a microplate reader at a wavelength of 450 nm.

### 2.4 Clone formation assay

The transfected cells were seeded in a 6-well plate and cultured for 1–2 weeks until the clones were visible. The medium was removed, and the cells were carefully washed twice with 2 mL of PBS. After the PBS was air-dried, the clones were fixed with 4% paraformaldehyde for 30 min at room temperature and then stained with 0.1% crystal violet dye for 30 min at room temperature. After dyeing, the dye was cleaned and then the number of clones was counted.

### 2.5 Flow cytometric analysis of apoptosis

The apoptosis of Weri-RB1 cells was evaluated by using an Annexin V-FITC Apoptosis Detection Kit (BD Biosciences) and PI staining according to the manufacturer’s instructions. After 48 h of transfection, the cells were digested with trypsin without EDTA, and centrifuged at 3,000*g* for 5 min at room temperature. About 3 mL PBS was added to resuspend and wash the cell precipitation. Subsequently, the cells were centrifuged at 3,000*g* for another 5 min at room temperature. 1× binding buffer was added to resuspend the cells, and the cell density was adjusted to 1–5 × 10^6^/mL. About 100 μL of the cell suspension was added into a 5 mL flow cytometry tube, and mixed with 5 μL Annexin V/FITC, and incubated in the dark for 5 min at room temperature. Then, 10 μL of the PI dye solution and 400 μL of PBS was added, and the apoptosis of Weri-RB1 cells in each group was analyzed by flow cytometer (FACS Calibur; BD Biosciences, Franklin Lakes, NJ). FlowJo software was used to analyze the flow cytometry outcomes.

### 2.6 Western blot analysis

After 48 h of transfection, the cells were lysed with RIPA lysate (Pierce, USA) in ice for 10 min, and the total protein was extracted. The concentration of protein was measured with the BCA protein assay kit (Pierce, USA). The protein samples were separated by SDS polyacrylamide gel electrophoresis and transferred into polyvinylidene difluoride membranes (PVDF; Millipore, Billerica, MA). The membranes were blocked with 5% skim milk for 1 h at room temperature and incubated overnight at 4°C with primary antibodies. The primary antibodies are as follows: anti-Bax (50599-2-Ig), anti-Bcl2 (12789-1-AP), anti-caspase3 (19677-1-AP), anti-p53 (60283-2-Ig), anti-GAPDH (60004-1-Ig), anti-AKT (10176-2-AP), anti-p-AKT (Ser473, 66444-1-Ig), anti-mTOR (66888-1-Ig), and anti-p-mTOR (Ser2448, 66888-1-Ig) were obtained from PTG, and anti-p70 (ab184551), anti-p-p70 (Ser371, ab109393), and anti-TRIB1 (ab137717) were obtained from Abcam. Following this, the secondary antibody (HRP-conjugated Goat Anti-Rabbit IgG, SA00001-2; and HRP-conjugated Goat Anti-Mouse IgG, SA00001-1, PTG) was applied and incubated with the membrane for 1 h at room temperature. Finally, the proteins were visualized with enhanced chemiluminescence reagents (Pierce, USA). The QUANTITY ONE software (Bio-Rad Laboratories, Inc.) was used to scan and calculate the relative expression quantity of each protein.

### 2.7 Double luciferase assay

The TRIB1 3′UTR region was cloned into the pmiR GLO vector, and the binding region of TRIB1 3′UTR to miR-513b-5p was mutated. TRIB1-3′UTR-WT and TRIB1-3′UTR-MUT plasmids were constructed and co-transfected into 293T cells with a miR-513b-5p mimics or negative control (NC) using Lipofectamine 2000 (Invitrogen), respectively. After 48 h, the Dual Luciferase Assay System Kit (Promega, WI, USA) was employed to analyze the activity of the luciferase.

### 2.8 Statistical analysis

GraphPad Prism 6.0 was used for data analysis (GraphPad Software Inc., San Diego, CA). All data were presented as the mean ± standard error of the mean (SEM). The differences were evaluated using one-way ANOVA followed by Tukey’s *post hoc* test for multiple comparisons and the Mann–Whitney *U*-test for pair-wise comparisons. A *P*-value less than 0.05 was considered to indicate a statistically significant difference.

## 3 Results

### 3.1 miR-513b-5p inhibits the proliferation of Weri-RB1 cells *in vitro*

**Figure 1:**
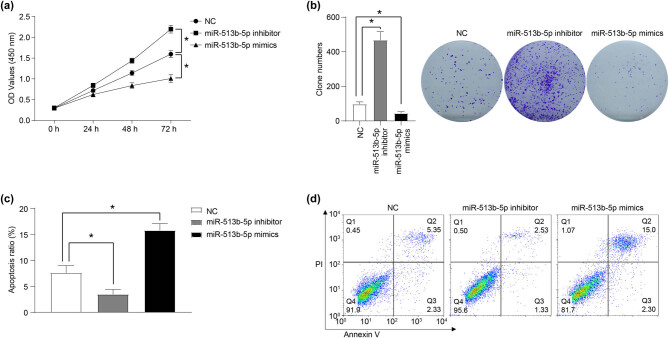
miR-513b-5p inhibits the proliferation and promotes the apoptosis of Weri-RB1 cells *in vitro*. The role of miR-513b-5p mimics and inhibitors in the proliferation, clone formation, and apoptosis of Weri-RB1 cells was detected by CCK-8 (a), clone formation (b), and flow cytometry (c and d) assays. The differences were evaluated using the Mann–Whitney *U*-test for pair-wise comparisons. *P < 0.05. Each group of a includes six samples, each group of b–d includes three samples. All experiments were repeated three times independently, and the individual experiments were performed 1 week apart.

In order to investigate the role of miR-513b-5p on the proliferation of Weri-RB1 cells, CCK-8 and clone formation assays were performed. The results of the CCK-8 assay showed that compared with the NC group, miR-513b-5p mimics inhibited the proliferation of Weri-RB1 cells, while the miR-513b-5p inhibitor promoted cell proliferation (Figure 1a, *P* < 0.05). In addition, the results of the clone formation experiment were consistent with the results of the CCK-8 assay. The number of cell clones formed in the miR-513b-5p mimics group was significantly lower than that in the NC group, while the clone numbers in the miR-513b-5p inhibitor group were decreased (Figure 1b, *P* < 0.05).

### 3.2 miR-513b-5p promotes the apoptosis of Weri-RB1 cells *in vitro*

**Figure 2:**
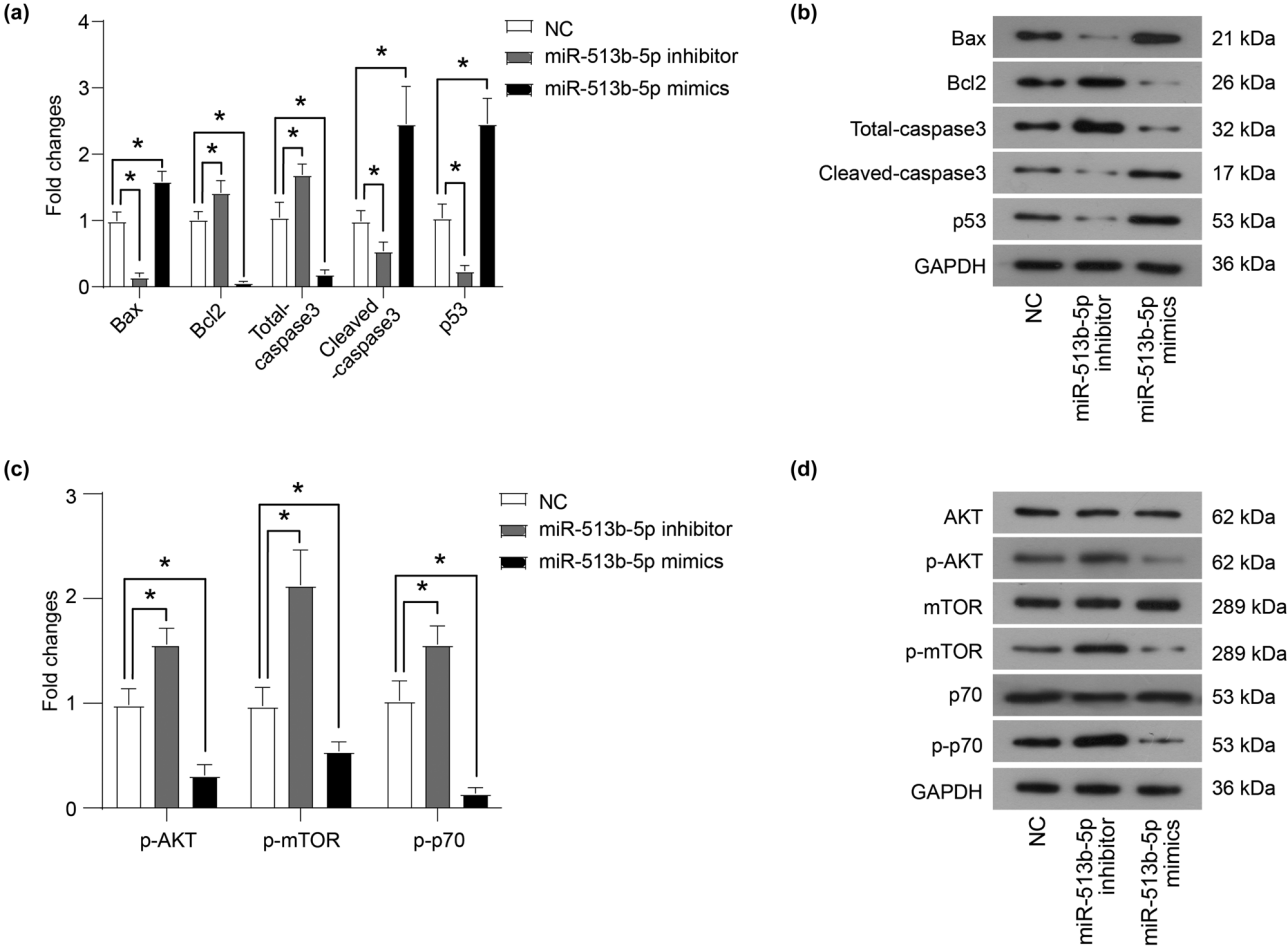
miR-513b-5p inhibits the activation of the AKT/mTOR signaling pathway. (a and b) The protein expression of key proteins related to apoptosis was measured using a western blot. (c and d) The protein expression of key proteins in the AKT/mTOR signaling pathway was measured using western blot. The differences were evaluated using the Mann–Whitney *U*-test for pair-wise comparisons. *P < 0.05. Each group of (b and d) includes three samples. All experiments were repeated three times independently, and the individual experiments were performed 1 week apart.

The role of miR-513b-5p on the apoptosis of Weri-RB1 cells was detected using flow cytometry and western blot. The results showed that the apoptosis ratio in the miR-513b-5p mimics group was significantly higher than that in the NC group, while the apoptosis ratio in the miR-513b-5p inhibitor group was decreased (Figure 1c and d, *P* < 0.05). In addition, after miR-513b-5p mimics were transfected, the protein level of anti-apoptotic protein Bcl2 was decreased, while the protein level of pro-apoptotic protein Bax, cleaved-caspase3, and p53 was increased (Figure 2a and b, *P* < 0.05). Furthermore, after transfection of miR-513b-5p inhibitor, the expression of anti-apoptotic protein Bcl2 increased, and the expression of pro-apoptotic protein Bax, cleaved-caspase3, and p53 decreased (Figure 2a and b, *P* < 0.05).

### 3.3 miR-513b-5p inhibits the activation of the AKT/mTOR signaling pathway

The effect of miR-513b-5p on the AKT/mTOR signaling pathway activation was determined by western blot. The results showed that miR-513b-5p mimics inhibited the phosphorylation of AKT, mTOR, and p70 (Figure 2c and d, *P* < 0.05). In contrast, the miR-513b-5p inhibitor increased the phosphorylation levels of AKT, mTOR, and p70 (Figure 2c and d, *P* < 0.05).

### 3.4 miR-513b-5p can bind to TRIB1 mRNA and regulate its protein expression

**Figure 3:**
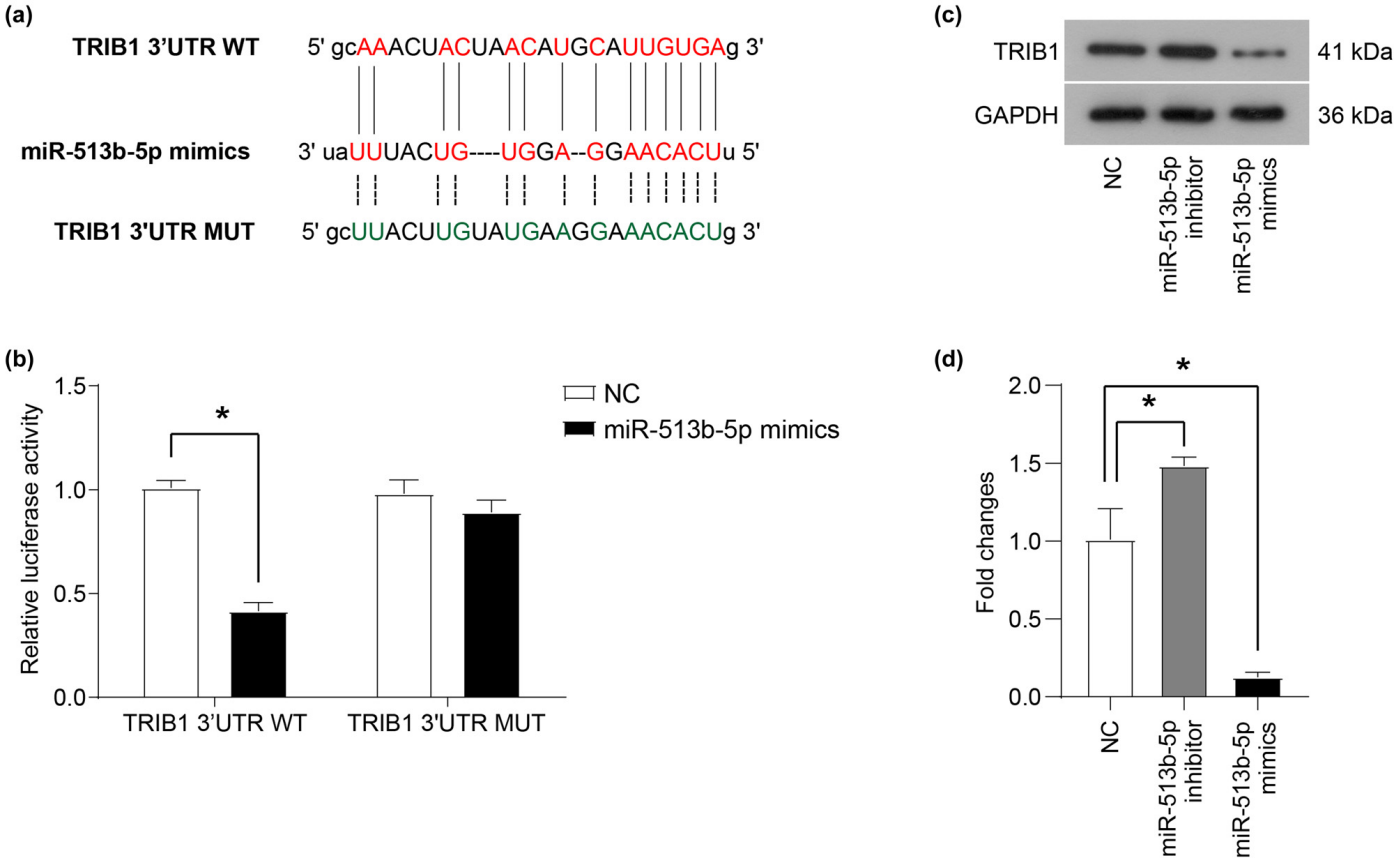
miR-513b-5p can bind to TRIB1 mRNA and regulate its protein expression. (a) The TargetScan software predicted the direct binding site of miR-513b-5p and the 3′UTR region of TRIB1 mRNA. (b) The relative expression of luciferase and direct binding of miR-513b-5p to the 3′UTR region of TRIB1 mRNA was determined by using a double-luciferase assay. (c and d) The protein expression of TRIB1 was measured using a western blot. The differences of (b) were evaluated using one-way ANOVA followed by Tukey’s *post hoc* test, and the Mann–Whitney *U*-test for (d). *P < 0.05. Each group of (b and d) includes three samples. All experiments were repeated three times independently, and the individual experiments were performed 1 week apart.

The TargetScan software predicted the direct binding site of miR-513b-5p and 3′UTR region of TRIB1 mRNA (Figure 3a). Subsequently, the direct binding of miR-513b-5p to the 3′UTR region of TRIB1 mRNA was determined by using a double-luciferase assay. As shown in Figure 3b, the co-transfection of miR-513b-5p mimics and pmiR-GLO-TRIB1-3′UTR-WT recombinant plasmid significantly reduced the expression of luciferase, while the co-transfection of miR-513b-5p mimics and pmiR-GLO-TRIB1-3′UTR-MUT recombinant plasmid did not significantly affect the level of luciferase. This result revealed that miR-513b-5p could directly bind to the 3′UTR region of the TRIB1 mRNA through the predicted site. In addition, the miR-513b-5p mimic inhibited the protein expression of TRIB1, while the miR-513b-5p inhibitor up-regulated the expression of the TRIB1 protein (Figure 3c and d, *P* < 0.05). In summary, miR-513b-5p can bind to the 3′UTR region of TRIB1 mRNA and regulate its protein expression.

### 3.5 TRIB1 promotes the proliferation and inhibits the apoptosis of Weri-RB1 cells *in vitro*

**Figure 4:**
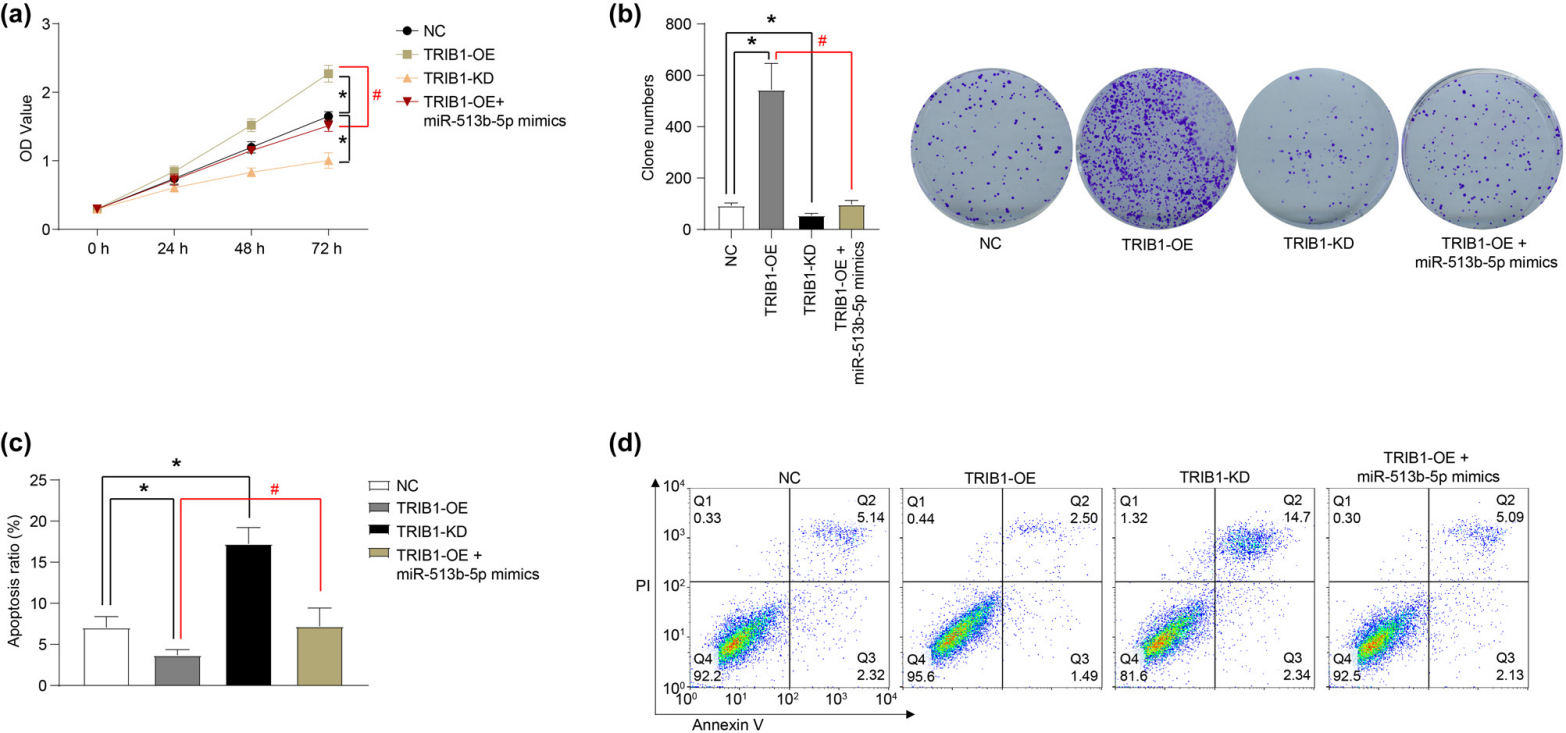
TRIB1 promotes proliferation and inhibits the apoptosis of Weri-RB1 cells *in vitro*. The role of TRIB1 overexpression (TRIB1-OE) and TRIB1 knockdown (TRIB1-KD) in the proliferation, clone formation, and apoptosis of Weri-RB1 cells was detected by CCK-8 (a), clone formation (b), and flow cytometry (c and d) assays. The differences were evaluated using the Mann–Whitney *U*-test for pair-wise comparisons. *P < 0.05, compared to NC; ^#^ *P* < 0.05, compared to TRIB1-OE. Each group of (a) includes six samples, each group of (b and d) includes three samples. All experiments were repeated three times independently, and the individual experiments were performed 1 week apart.

**Figure 5:**
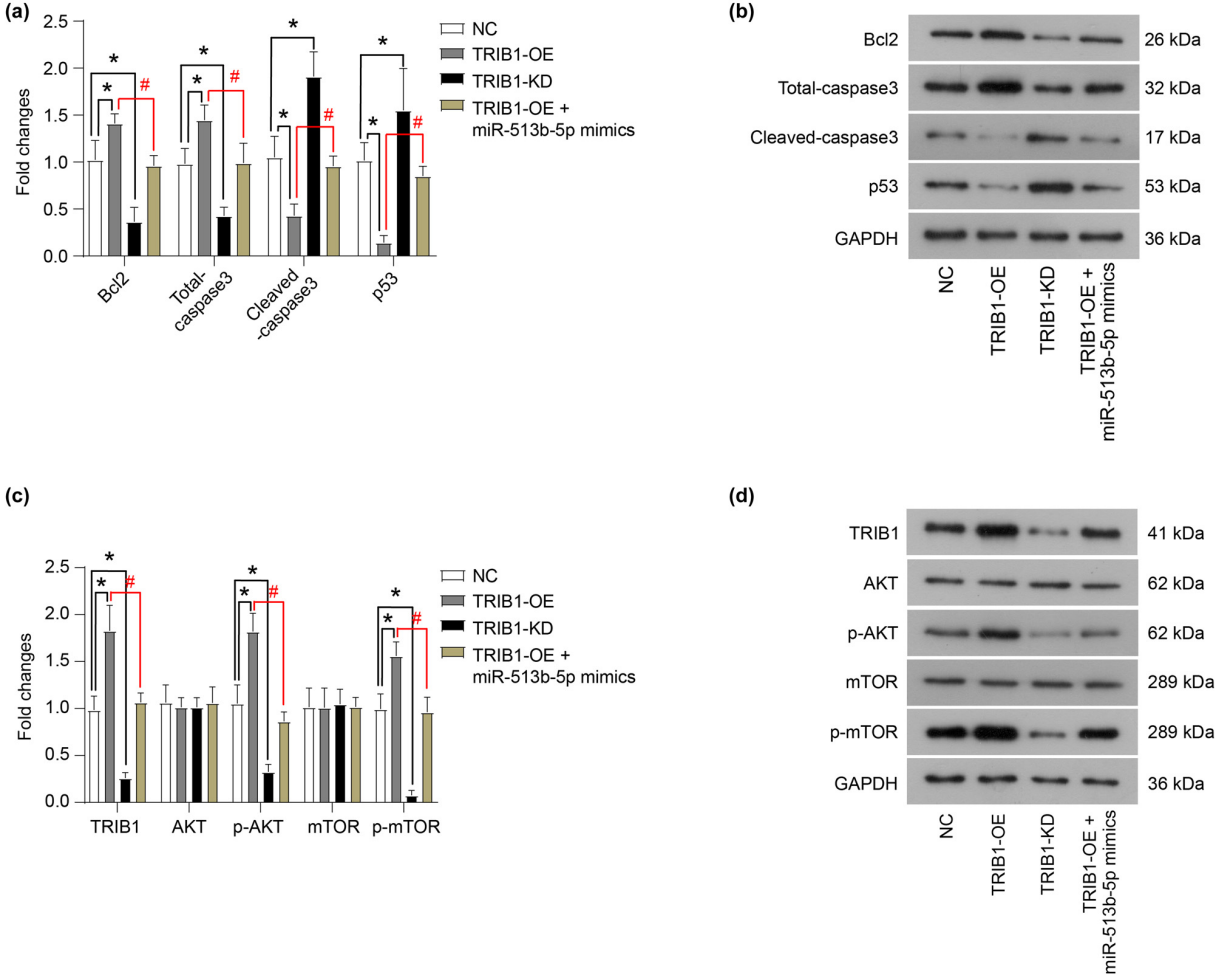
TRIB1 promotes the activation of the AKT/mTOR signaling pathway. (a and b) The protein expression of key proteins related to apoptosis was measured using western blot. (c and d) The protein expression of key proteins in the AKT/mTOR signaling pathway was measured using western blot. The differences were evaluated using the Mann–Whitney *U*-test for pair-wise comparisons. *P < 0.05, compared to NC; ^#^ *P* < 0.05, compared to TRIB1-OE. Each group of (b and d) includes three samples. All experiments were repeated three times independently, and the individual experiments were performed 1 week apart.

The effect of TRIB1 on the proliferation and apoptosis of Weri-RB1 cells was determined. As shown in Figure 4a and b, the overexpression of TRIB1 (TRIB1-OE) promoted the proliferation and clone formation of Weri-RB1 cells, while the knockdown of TRIB1 (TRIB1-KD) inhibited the proliferation and clone formation of Weri-RB1 cells. In addition, the overexpression of TRIB1 inhibited the apoptosis of Weri- RB1 cells (Figure 4c and d), upregulated the protein expression of Bcl2, and downregulated the protein expression of cleaved-caspase3 and p53 (Figure 5a and b). The knockdown of TRIB1 induced apoptosis of Weri-RB1 cells (Figure 4c and d), downregulated the protein expression of Bcl2 and upregulated the protein expression of cleaved-caspase3 and p53 (Figure 5a and b). Furthermore, the miR-513b-5p mimic could neutralize the role of TRIB1 overexpression on the proliferation and apoptosis of Weri-rb1 cells (Figures 4a–5b, *P* < 0.05).

In addition, the overexpression of TRIB1 promoted the phosphorylation of AKT and mTOR, while the knockdown of TRIB1 decreased the phosphorylation levels of AKT and mTOR (Figure 5c and d, *P* < 0.05). Furthermore, miR-513b-5p can counteract the effect of TRIB1 overexpression on the AKT and mTOR phosphorylation (Figure 5c and d, *P* < 0.05).

## 4 Discussion

RB is a malignant ocular tumor in infants with a high blindness rate. In China, approximately 1,000 children are diagnosed with RB every year [15]. Moreover, about 5% of children’s blindness is caused by RB [16]. Therefore, it is of great significance to investigate the pathogenesis and therapeutic targets of RB.

It is well known that miRNAs are involved in the pathogenesis of various human malignancies. In this study, we found that miR-513b-5p inhibited the proliferation and promoted apoptosis of Weri-RB1 cells *in vitro*. The results of this research are consistent with previous studies on the role of miR-513b-5p in NSCLC [8], embryonic testicular cancer [13], gastric cancer [10], and osteosarcoma [17] cells, but are contrary to the role of miR-513b-5p in cervical cancer [11] and ovarian cancer [12]. This may be due to the uniqueness of the tumor type.

TRIB1 (tribbles homologue 1) is a member of the Tribbles family, which is initially found to modulate string/cdc25 in Drosophila morphogenesis [18]. TRIB1 has been reported to be associated with the occurrence and development of a variety of tumors, including leukemia [19], hepatocellular carcinoma [20], colorectal cancer [21], and prostate cancer [22]. It has been revealed that TRIB1 is highly expressed in prostate cancer and follicular thyroid cancer, and promotes the proliferation, survival, and tumor growth of cancer cells. In this article, we determined that TRIB1 is the direct binding target of miR-513b-5p and detected that its overexpression contributed to the proliferation and cloning of Weri-RB11 cells, but inhibited their apoptosis. Our findings are consistent with prevenient conclusions about the role of TRIB1 in malignancies, suggesting that TRIB1 may be an oncogene.

The dual-luciferase assay confirmed that TRIB1 is the direct binding target of miR-513b-5p. In addition, the results of western blot showed that miR-513b-5p inhibited the protein expression of TRIB1. Meanwhile, this study also clarified that miR-513b-5p/TRIB1 can regulate the phosphorylation of the PI3K signaling pathway. The overexpression of miR-513b-5p inhibited the phosphorylation of AKT, mTOR, and p70, while TRIB1 played the opposite role. However, there are many shortcomings in our experiment, such as the lack of *in vivo* experiments and the relatively single target and action mechanism. Further studies will be conducted.

In conclusion, our study suggested that miR-513b-5p inhibited the expression of TRIB1 and exerted a tumor suppressor gene in Weri-RB1 cells. We demonstrated for the first time the role and the interaction of miR-513b-5p and TRIB1 in Weri-RB1 cells. It is speculated that miR-513b-5p and TRIB1 may be the biomarkers and/or key targets for clinical diagnosis and treatment of RB in the future.
